# Ultrasound evaluation of diaphragm motion in BAG-3 myofibrillar myopathy: A case report

**DOI:** 10.1097/MD.0000000000028484

**Published:** 2022-01-07

**Authors:** Liqiong Zhan, Lan Lv, Xinyuan Chen, Xiang Xu, Jun Ni

**Affiliations:** a Department of Rehabilitation Medicine, First Affiliated Hospital of Fujian Medical University, Fuzhou, China; b Department of Ultrasound Medicine, First Affiliated Hospital of Fujian Medical University, Fuzhou, China.

**Keywords:** BAG-3, diaphragmatic paralysis, myofibrillar myopathy, ultrasound

## Abstract

**Rationale:**

Mutations in Bcl-2-associated athanogene-3 (BAG-3) can cause a rare subtype of myofibrillar myopathies (MFMs), characterized by progressive muscle weakness, cardiomyopathy, and severe respiratory insufficiency in childhood. Little is known about diaphragmatic function in BAG-3 MFM. To our knowledge, this is the first case report of detailed evaluation of diaphragmatic function with ultrasound in BAG-3 MFM.

**Patient concern:**

We describe the case of a 15-year-old girl who complained of fever and shortness of breath. Diaphragmatic sonography revealed bilateral diaphragmatic paralysis. Shortness of breath progressed to respiratory failure approximately 3 months later.

**Diagnosis:**

A neurologist was consulted and genetic sequencing identified a p.Pro209Leu mutation in BAG-3, yielding diagnosis of BAG-3 MFM leading to bilateral diaphragmatic paralysis.

**Interventions:**

Respiratory muscle training and long-term mechanical ventilation.

**Outcomes:**

It is quite unfortunate for this patient to have a poor prognosis due to the lack of effective treatment for this genetic disorder.

**Lessons:**

This case provides more clinical information for this rare disease which may cause severe diaphragm pathological damage leading to respiratory failure in BAG3 MFM and a future study with a systematic evaluation of a greater number of patients will be necessary to characterize this population.

## 1 Introduction

The development of diaphragmatic paralysis may be attributed to neuromuscular diseases, trauma, iatrogenic diseases, tumor compression, infection/inflammation, and idiopathic causes. Myofibrillar myopathies (MFMs) are a group of muscle diseases with genetic and clinical heterogeneity that are characterized by myofibrillar dissolution, Z-disk decomposition, accumulation of degradation products, and abnormal protein expression.^[1]^ To date, pathogenic mutations in different genes have been found to be associated with MFMs. They are divided into different subtypes based on the different mutant genes. Among them, BCL2-associated athanogene 3 (BAG3) gene mutation is thought to induce a rare MFM subtype VI with clinical features of rapidly progressive limb and axial muscle weakness, cardiomyopathy, and respiratory insufficiency in childhood. Three cases of this subtype were reported for the first time in the *Annals of Neurology* in 2009.^[2–13]^ Respiration function is often involved, providing a severe restrictive pattern that leads to respiratory failure. Here, we report a rare case of bilateral paralysis of the diaphragm detected by ultrasound in a patient with BAG-3 mutation-related MFMs. Informed consent was obtained from the patient and her families.

## 2 Case report

**Figure 1:**
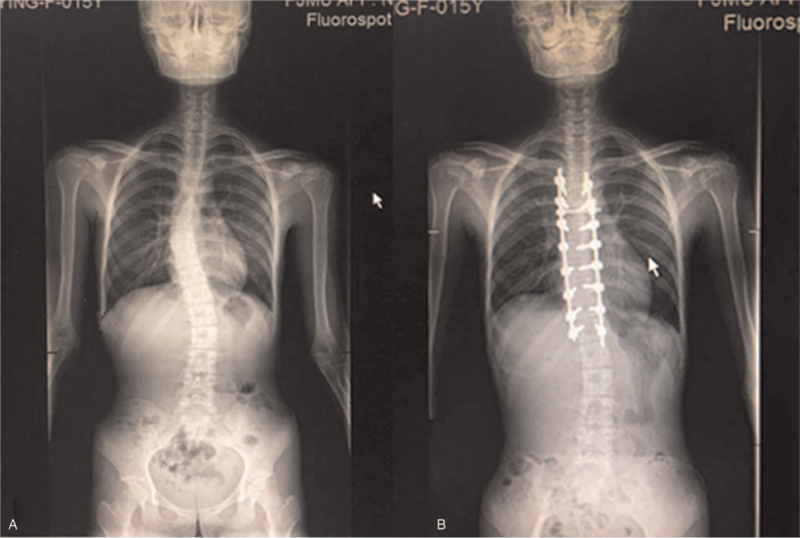
A, Preoperative spinal X-ray film. B, Postoperative spinal X-ray film.

A 15-year-old girl presented to the Emergency Department of the Affiliated Hospital of Putian University due to fever and shortness of breath on April 1, 2020. She had a medical history of “surgical correction of scoliosis” in the First Affiliated Hospital of Fujian Medical University due to “scoliosis” 3 months and 10 days earlier (December 20, 2019) (Fig. 1). Before the fever, the family members had found that the patient's bilateral neck and shoulder muscles twitched with breathing while sleeping at night, with dyspnoea and fatigue after long-distance walking. After admission, routine blood testing was taken and the results are summarized in Table 1 (the first time). Chest computed tomography showed inflammation in both the lungs. The patient was diagnosed with viral upper respiratory tract infection, pneumonia, viral myocarditis, and cardiac insufficiency. There was no fever after 2 weeks of anti-infectious therapy. Reexamination via lung computed tomography showed that the inflammation was alleviated, but the symptoms of shortness of breath were more aggravated than before.

**Table 1:** Blood test results of the patient at different times.

	WBC, /mm^3^	N, %	CRP, mg/L	Pro-BNP, pg/mL	TNI, ng/mL	CK-MB, U/L
1st time	11,140	85.7	88.89	3789	0.12	NA
2nd time	7780	73.7	<5	4048	0.24	207
3rd time	NA	NA	NA	8424	0.23	NA
4th time	7280	67.9	38.91	4920	0.17	223
5th time	10,690	78.3	<5	7288	0.29	NA

**Figure 2:**
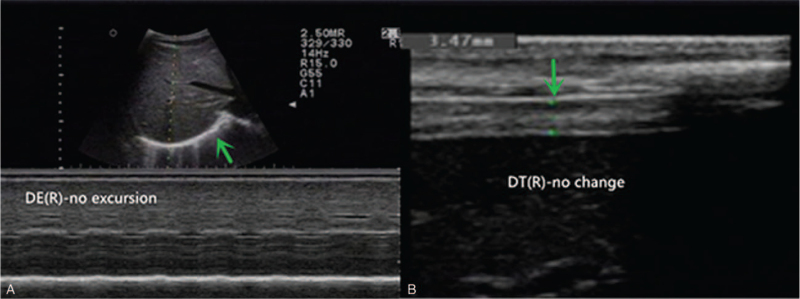
Ultrasonographic examination of the right diaphragm. The hyperechoic window of the right diaphragm in the 7th costal space could be observed at the distal end of the liver. A, The diaphragm excursion (DE) was observed by M-type ultrasound with no observed diaphragm movement (green arrow). B, The right diaphragm thickness (DT) (3.5 mm) was observed through B-type ultrasound, with no change during inspiration (green arrow).

**Figure 3:**
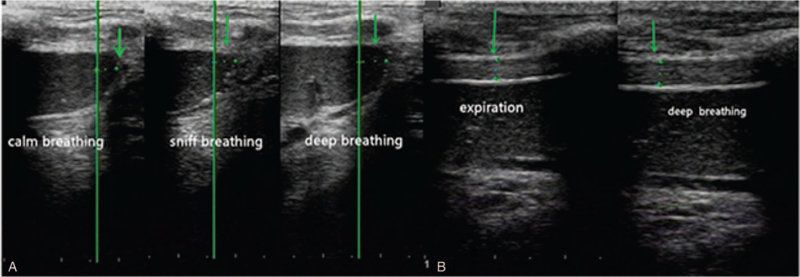
Ultrasonographic examination of the left diaphragm. A, The lower pole of the spleen moved 3.4 mm with calm breathing, 4.7 mm with deep breathing, and 3.7 mm with sniff breathing (green arrow). B, The left DT was observed by B-type ultrasound. The left DT was 2.9 mm at the end of expiration and 3.4 mm at the end of deep breathing (green arrow).

The patient was immediately transferred to the Department of Cardiology of the First Affiliated Hospital of Fujian Medical University on April 16, 2020. Blood tests were performed for the second time (Table 1). Colour Doppler ultrasound showed a small left heart, thickening of the left ventricular wall, decreased left ventricular diastolic function, and no abnormality in the left ventricular ejection fraction value. The patient was then diagnosed with viral myocarditis and cardiac insufficiency and provided myocardial nutrition, heart rate control, and diuretic therapies. The patient underwent pulmonary function tests after improvement of dyspnoea. The pulmonary function test showed a forced vital capacity (FVC) of 0.69 L or 21.8% of the predicted value, forced expiratory volume in 1 s (FEV_1_) of 0.61 L or 22.9% of the predicted value and FEV_1_/FVC ratio of 89.11%. Diaphragm ultrasonography revealed right diaphragm paralysis with no actual excursion in the M-mode or thickness change in the B-mode (Fig. 2). There was no clear display of left-diaphragm activity and craniocaudal displacement of the lower pole of the spleen of 3.4 mm during calm breathing, 4.7 mm during deep breathing, and 3.7 mm during sniff breathing. The thickness of the left diaphragm was 2.9 mm at the end of expiration and 3.4 mm at the end of deep breathing (Fig. 3). Consultation with a cardiopulmonary physiotherapist was recommended.

The cardiopulmonary physiotherapist checked the patient and found the following: shallow, fast breathing, with visible activity of the accessory respiratory muscle; grade 3–4/5 muscle strength of the limbs. The patient was provided breathing training (inspiratory muscle strength training and air holding, auxiliary airway clearance training) by the cardiopulmonary physiotherapists to improve her breathing. However, the cause of diaphragmatic paralysis could not be determined before the patient was discharged on April 24, 2020.

**Figure 4:**
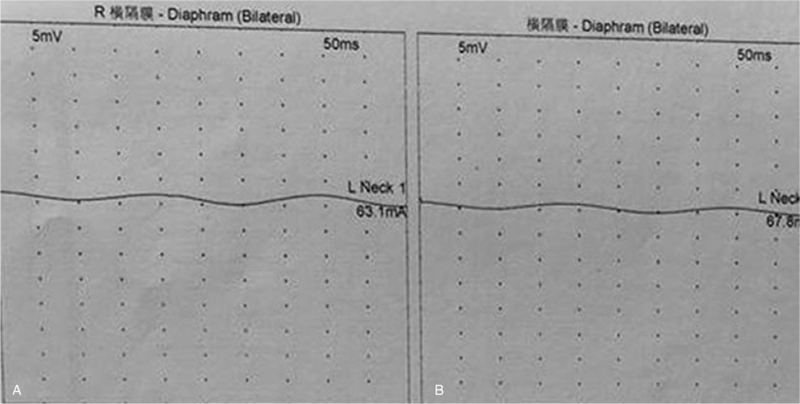
Nerve conduction studies showed no response to nerve stimulation in the right (A) and left (B) diaphragms.

Twenty-eight days after discharge from the First Affiliated Hospital of Fujian Medical University (May 22, 2020), the patient was referred to another hospital, and no obvious abnormality was found on brain magnetic resonance imaging. Cervical and thoracic magnetic resonance imaging indicated cervical dysplasia and postoperative changes in T5-T12. Electromyography showed that the amplitude of somatosensory-evoked potential (SSEP) in the left median nerve was decreased, the latency was prolonged; the SSEP of the right median nerve was not evoked after repeated attempts, and there was no SSEP of the bilateral tibial nerve after multiple repetitions. The conduction velocity of the bilateral phrenic nerve was not elicited (Fig. 4), and the cause of diaphragmatic dysfunction remains unclear.

Two months and eight days after discharge from the First Affiliated Hospital of Fujian Medical University (July 2, 2020), the patient developed worsening shortness of breath again with polyserous effusion, was unable to lie flat, and showed facial and lower limb edema and respiratory failure. The patient returned to the Department of Cardiology of the hospital again. The fourth blood test result is summarized in Table 1. Full abdominal color Doppler ultrasound showed liver congestion, and blood gas analysis showed type II respiratory failure. The patient was provided with anti-infection, cardiotonic therapy, puncture and drainage of effusion, and ventilator-assisted breathing. Following consultation with a physician in the Department of Neurology, the patient was diagnosed with MFM subtype VI after the discovery of BAG3: c.626C > T:p.P209L by whole-exome sequencing.

## 3 Discussion

The majority of human BAG-3-related MFMs are attributed to the heterozygous mutation p.Pro209Leu (c.626C > T) in exon 3. BAG-3 has been recognized in a series of cellular processes, such as development, apoptosis, and autophagy, as well as acting as cytoskeleton tissue and in cell adhesion and movement. Although BAG3 is expressed in various tissues, it is particularly abundant in the bone and myocardium under mechanical strain.^[14]^ BAG3 deficiency can lead to fulminant myopathy and early death in mice.^[15]^ The clinical manifestations are rapid progressive weakness cardiomyopathy, axonal multiple neuropathy, severe respiratory insufficiency, and possible scoliosis in the first or second decade of life, which is consistent with the phenotype of our patient. A diagnosis of the patient can be confirmed by reviewing both the clinical and gene analysis results.

BAG-3 p.Pro209Leu (c.626C > T) mutation-induced MFM subtype VI is quite rare clinically, with only 19 patients reported at present, of whom 17 patients already had respiratory insufficiency at the time of report, and most required mechanical ventilation support.^[2–13]^ However, these patients generally did not undergo further evaluation of their respiratory function. Two patients were reported to have bilateral diaphragmatic paralysis, but no diagnostic details have been reported.^[2,11]^ One patient underwent electromyography of the phrenic nerve and diaphragm, with no response to phrenic nerve stimulation and with chronic denervation of the diaphragm, which was consistent with the results of phrenic nerve electromyography in our case.^[4]^

Diaphragmatic paralysis can be diagnosed using various methods. Pulmonary function testing is a useful method for screening. The reduction of vital capacity in the patient from the sitting position to the supine position by ≥20% or more may indicate diaphragmatic weakness.^[16]^ Fluoroscopy combined with sniff tests can be used to diagnose diaphragm paralysis but has a limited range of applications owing to radiation exposure to patients.^[17]^ Furthermore, electromyography can also be used to diagnose diaphragmatic paralysis, but the skin electrode may be affected by the placement position, and needle electromyography is invasive.^[16]^ Measurement of the maximum trans-diaphragmatic pressure during the maximum inspiratory effort or phrenic nerve stimulation is considered to be reliable for the diagnosis of diaphragmatic paralysis; however, it requires placement of oesophageal and gastric balloon catheters, which are invasive and expensive.^[18]^ Bedside ultrasound is also a useful technique for evaluation. The degree of diaphragm thickening from FRC to TLC (Δtdi%) was expressed as the percentage change in tdi relative to tdi at FRC (i.e., Δtdi%/tdi FRC X100). A patient can be diagnosed with diaphragmatic paralysis when the thickening of the diaphragm (ΔTdi%) by ultrasound is <20%.^[19,20]^ In our patient, ultrasound evaluation showed that the patient had almost no thickness change in the right diaphragm, and the ΔTdi% of the left diaphragm was <20%. In addition, diaphragmatic range of motion is also a common choice for evaluating diaphragmatic function. It is more frequently used as a qualitative assessment because of the possibility of being affected by abdominal tissue. In our patient, there was no movement of the diaphragm on the right side, and a clear image could not be acquired on the left side because of the interference of gastric and intestinal gas. As reported by Toledo et al,^[21]^ the range of movement of the lower spleen and splenic hilum during breathing was used to reflect the dome of the left diaphragm, and there was an extremely poor motion of the left spleen. In our patient, the diagnosis of bilateral diaphragmatic paralysis was confirmed by electromyographic and ultrasonographic results.

Diagnosis of bilateral diaphragmatic paralysis is relatively easy, and the greatest difficulty is generally the clarification of the etiology at times. Our patient also underwent surgical correction of scoliosis and heart damage, leading to confusion in the diagnosis. It took several months from the discovery of bilateral diaphragmatic paralysis by ultrasound to the final genetic diagnosis. The treatments for bilateral diaphragmatic paralysis include respiratory muscle training and long-term mechanical ventilation. Phrenic nerve pacing has been reported for diaphragmatic paralysis caused by cervical spinal cord injury and amyotrophic lateral sclerosis; however, its effectiveness and safety have not been proven.^[22]^ Among the currently reported cases, patients eventually died of cardiopulmonary failure.

To our knowledge, our case involving a patient with bilateral diaphragmatic paralysis caused by rare BAG-3-related MFMs observed for the first time by ultrasound is unique. The value of our case is that the reported data may provide more clinical information for this rare disease. Our case also indicates that the diaphragm may exhibit more severe pathological damage relative to the myocardium and peripheral skeletal muscles. Additional studies are required to confirm this possibility.

## Author contributions

**Conceptualization**: Liqiong Zhan, Lan Lv

**Formal analysis:** Liqiong Zhan

**Investigation**: Liqiong Zhan, Lan Lv, Xiang Xu, Xinyuan Chen, Jun Ni

**Methodology**: Liqiong Zhan, Lan Lv

**Validation:** Liqiong Zhan, Lan Lv.

**Visualization**: Lan Lv, Xiang Xu, Xinyuan Chen.

**Writing – original draft**: Liqiong Zhan, Lan Lv, Xiang Xu, Xinyuan Chen

**Writing – review & editing**: Liqiong Zhan, Lan Lv, Xiang Xu, Xinyuan Chen, Jun Ni

## Correction

When originally published, the funding information, the Startup Fund for scientific research, Fujian Medical University (Grant number: 2017XQ1088), was not included. This information has been added to the article footnote.
